# Rod function deficit in retained photoreceptors of patients with class B *Rhodopsin* mutations

**DOI:** 10.1038/s41598-020-69456-3

**Published:** 2020-07-28

**Authors:** Artur V. Cideciyan, Samuel G. Jacobson, Alejandro J. Roman, Alexander Sumaroka, Vivian Wu, Jason Charng, Brianna Lisi, Malgorzata Swider, Gustavo D. Aguirre, William A. Beltran

**Affiliations:** 1 Scheie Eye Institute, Department of Ophthalmology, Perelman School of Medicine, University of Pennsylvania, Philadelphia, PA 19104 USA; 2 Division of Experimental Retinal Therapies, Department of Clinical Sciences and Advanced Medicine, School of Veterinary Medicine, University of Pennsylvania, Philadelphia, PA 19104 USA

**Keywords:** Hereditary eye disease, Retinal diseases, Molecular medicine, Genetic predisposition to disease, Retina

## Abstract

A common inherited retinal disease is caused by mutations in *RHO* expressed in rod photoreceptors that provide vision in dim ambient light. Approximately half of all *RHO* mutations result in a Class B phenotype where mutant rods are retained in some retinal regions but show severe degeneration in other regions. We determined the natural history of dysfunction and degeneration of retained rods by serially evaluating patients. Even when followed for more than 20 years, rod function and structure at some retinal locations could remain unchanged. Other locations showed loss of both vision and photoreceptors but the rate of rod vision loss was greater than the rate of photoreceptor degeneration. This unexpected divergence in rates with disease progression implied the development of a rod function deficit beyond loss of cells. The divergence of progression rates was also detectable over a short interval of 2 years near the health-disease transition in the superior retina. A model of structure–function relationship supported the existence of a large rod function deficit which was also most prominent near regions of health-disease transition. Our studies support the realistic therapeutic goal of improved night vision for retinal regions specifically preselected for rod function deficit in patients.

## Introduction

Vision is initiated with the absorption of photons by opsin molecules located in the outer segment antenna of retinal photoreceptor cells. Resulting hyperpolarization of the photoreceptor plasma membrane activates inter-neuronal signaling pathways which reach the visual cortex culminating in perception. Any number of defects along the complex visual pathway can give rise to loss of vision. Monogenic defects causing inherited retinal diseases (IRDs) act primarily at retinal photoreceptors and provide an opportunity to understand detailed mechanism of vision loss. One of the most common IRDs is due to mutations in *Rhodopsin* (*RHO*) associated with autosomal dominant retinitis pigmentosa (adRP)^1–4^. *RHO* encodes the opsin molecules expressed only in rod photoreceptors which provide night vision. Thus, the primary consequence of the molecular defect in *RHO*-adRP is abnormal night vision. Day vision mediated by cone photoreceptors are often affected secondarily through non–cell-autonomous mechanisms. There are currently no approved treatments for *RHO*-adRP, but promising therapeutic approaches directed to the rod photoreceptors include knock-down-and-replace gene therapy^5^, anti-sense oligonucleotides^6^, and modification of proteasomal activity^7^.

The human *RHO*-adRP disease phenotype is well represented by only two classes of mutations^8–13^ despite the hypothesized existence of a variety of pathomechanisms *in vitro*^14,15^. Patients with Class A phenotype have severe, early and retina-wide loss of rod photoreceptors with residual visual function originating only from cone cells. In Class B *RHO* phenotypes on the other hand, loss of rods is limited to certain retinal regions, or sectors, but rods (and cones) are retained in neighboring regions. This phenotypic classification has been confirmed by independent investigators using direct measurement of rod and cone photoreceptor function^16^. In addition, clinical descriptions of “sector”, “regional” or “mild” forms of adRP with “recordable rod ERGs” can be assumed to describe Class B phenotype with retained rods, at least to a first approximation^17–27^. Taken together, the literature suggests that more than half of ~ 150 different *RHO* mutations may cause Class B phenotype^28^ and thus contain stages of disease, at least in some family members and at some ages, that are amenable for rod photoreceptor-directed treatments. In the current study, we examined structural and functional features of rods recorded serially in Class B *RHO*-adRP in order to define the spatiotemporal properties of slow disease progression. In addition, we report discovery of a large rod function deficit and we describe the spatial distribution of the dysfunctional photoreceptors which may be ideal targets for improved vision with efficacious treatments.

## Results

### Progressive vision loss in some retinal regions but stability in others

Class B *RHO*-adRP is a progressive disease. Cross-sectional studies of patients at different stages of disease have suggested a complex spatio-temporal pattern involving the interaction of altitudinal, pericentral and diffuse degenerations resulting in a region or sector of the retina that is substantially less affected compared to other regions^8–13^. Among the unresolved questions is whether different retinal locations have different onset of disease followed by invariant rates of degeneration (as previously proposed for *ABCA4*-disease^29,30^) or whether different regions have different progression rates or a complex combination of these features. Considering progression of *RHO*-adRP is thought to be slower than most other IRDs^31,32^, and Class B *RHO*-adRP phenotype is milder than Class A^8^, we made serial visual function measurements over an ultra-long interval of approximately 20 years in a cohort of patients to quantify progression.

**Figure 1:**
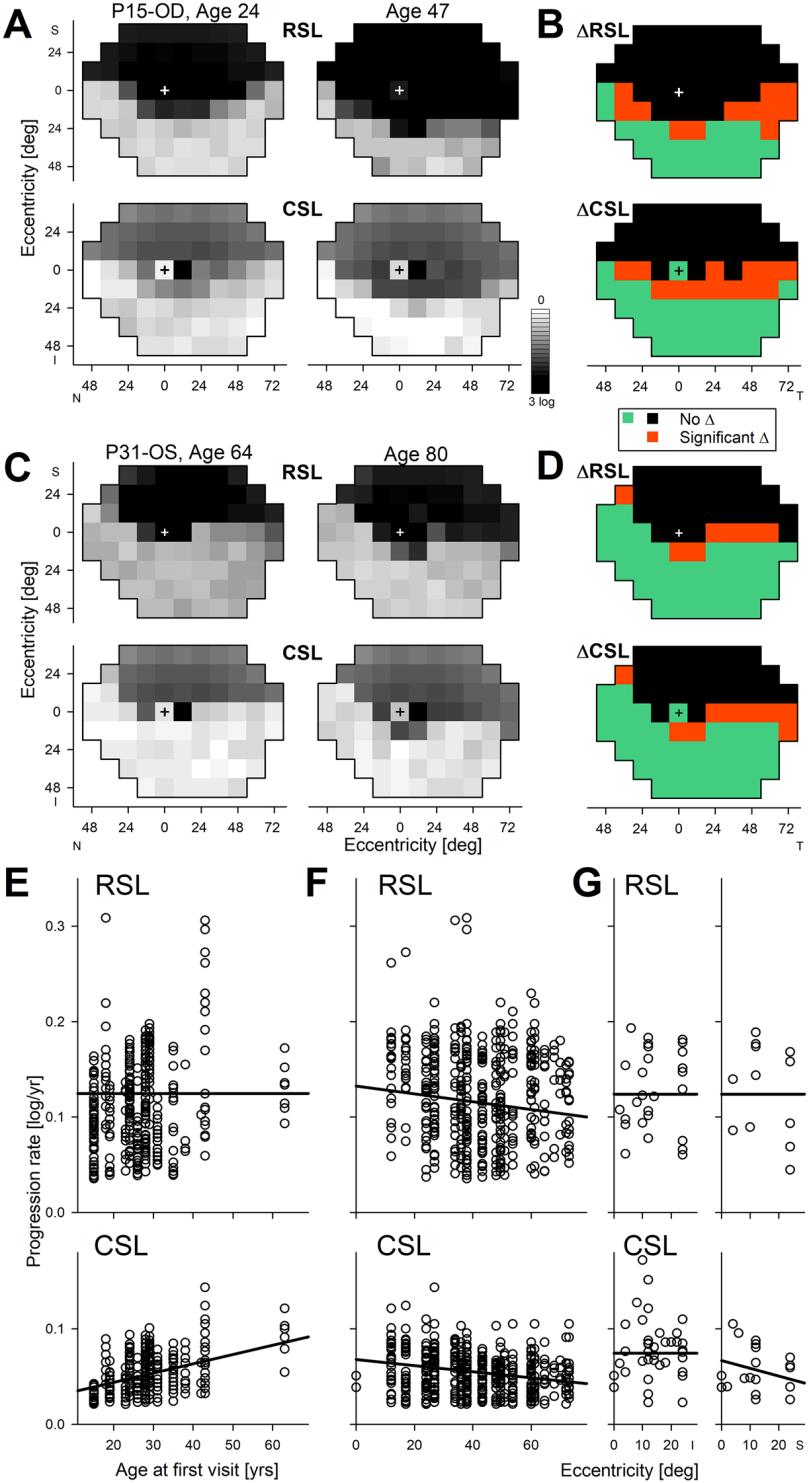
Slowly progressive loss of rod and cone vision in patients with Class B *RHO* mutations followed for approximately two decades. (**A**, **C**) Rod and cone sensitivity losses (RSL and CSL) across the visual field mapped in grayscale in a younger (**A**) and an older (**C**) representative patient. Serial measurements performed over intervals of approximately two decades. (**B**, **D**) Localization of significant changes (Δ) of RSL and CSL shown in panels A and C. Black, severe loss of sensitivity at both visits. Orange, mild to moderate losses of sensitivity at first visit that change more than the expected variability of the test. Green, no significant change. + , loci of foveal fixation. (**E**, **F**) Progression rates of RSL (upper) and CSL (lower) as a function of age at first visit (**E**) and as a function of eccentricity (**F**). (**G**) RSL and CSL progression rates along the central vertical meridian at higher spatial resolution obtained in a smaller cohort with serial long-term data. Solid lines (**E**–**G**) are linear regressions from a mixed-effects model (MEM). S, I, N, T, refer to superior, inferior, nasal and temporal visual field. Eccentricity is specified as degrees subtended from foveal fixation locus. Note, inferior visual field corresponds to superior retina, and temporal visual field to nasal retina.

Topographic distribution of rod and cone function across the full visual field in two representative patients with Class B *RHO*-adRP shows the classic “altitudinal” defect where vision is compromised in the superior visual field but relatively retained in the inferior visual field, and this can be true in younger (Fig. 1A) and older (Fig. 1C) subjects demonstrating heterogeneity of severity but homogeneity of pattern. More careful examination reveals that the altitudinal vision loss extends to the immediate pericentral area surrounding the fovea completely (Fig. 1A) or partially (Fig. 1C). Measurement of topographic rod and cone sensitivities across the visual field approximately 20 years later shows relatively minor changes (Fig. 1A,C). There are two likely causes for change: variability of the measurement, disease progression, or both. We took advantage of the very long interval and the size of the expected measurement variability^33^ to identify those locations where disease progression dominates (Fig. 1B,D, orange). The majority of locations with relatively retained rod and cone vision at the first visit surprisingly showed no evidence of progression despite the decades-long interval (Fig. 1B,D, green). And there were locations with severe vision loss already at the first visit that could not show further change (Fig. 1B,D, black). Locations with evidence of disease progression tended to be located near disease-health transitions and this was not unexpected based on the centripetal constriction of kinetic visual fields with increasing disease severity^11,34^; however, the paucity of progressing locations was very unexpected.

In the cohort with serial recordings over an ultra-long interval, there were 16 eyes of 16 patients with Class B *RHO*-adRP (Table S1). At first visit, mean age was 32 (range 15–63) years; last visit occurred on average 21 years later (range 10–28 years). Retinal loci with severe sensitivity loss at first visit (~ 30% of samples) were censored since progression would not be measurable and these locations would otherwise only contribute a floor effect artefactually reducing the overall progression rate. Retinal loci with mild and intermediate rod sensitivity loss (RSL) at first visit showed a nominal progression rate of 0.08 log/year. However, nearly one third of the samples demonstrated a magnitude of change that was still within test–retest variability^33^ despite the intervening two-decade interval. The remaining third of the samples showed substantial changes in sensitivity thus were thought to represent the retinal loci with true disease progression with a rate estimate of 0.13 log/year. The rate of rod progression did not vary with age (Fig. 1E, upper, *p* = 0.52, mixed-effects model, MEM) implying that once retinal disease is initiated at a location, the ensuing rate of progression is nearly invariant. The rate of rod progression vs. eccentricity across the retina showed tendency for greater progression in the perimacular region as compared to the periphery; the effect was small but statistically significant (Fig. 1F, upper, coefficient − 0.0004 (log/year)/deg, *p* < 0.0001, MEM).

Similar analyses performed at locations with mild to moderate cone sensitivity losses (CSL) at first visit showed a nominal progression rate of 0.04 log/year, which increased to 0.06 log/year when considering subset of locations demonstrating significant change. CSL progression rate showed a relationship with age (Fig. 1E, lower, coefficient 0.001 (log/year)/year of age, *p* = 0.007, MEM) implying secondary “bystander” cone disease to be more prominent in older patients compared to younger. CSL progression also showed a small but significant tendency for greater progression centrally (Fig. 1F, lower, coefficient − 0.0003 (log/year)/deg *p* < 0.0001, MEM), similar to RSL results.

From the macroscopic topography of rod and cone function sampled sparsely across the retina (Fig. 1A–F) we next zoomed into the central retina where microscopic changes to retinal structure could be quantified. We used serial measurements performed over the long term at higher spatial resolution along the central vertical meridian in a subset of 10 eyes of 8 patients together with more limited measurements in 8 eyes of 8 patients (Table S1) to estimate progression rates. For central retinal loci with mild to moderate sensitivity loss demonstrating significant change over the long interval, mean RSL progression rates were 0.12 and 0.12 log/year, respectively, for inferior and superior visual fields (*p* < 0.0001, MEM, Fig. 1G). Mean CSL progression rates were 0.075 and 0.06 log/year, respectively, for inferior and superior visual fields (*p* < 0.0001, MEM, Fig. 1G). For the superior visual field, there was minor but statistically significant tendency of greater progression more centrally (eccentricity coefficient − 0.0008 (log/year)/deg, *p* = 0.02, MEM, Fig. 1G). Overall, central progression rates were comparable to retina-wide rates (Fig. 1E–G).

### Retinal structural underpinnings of vision loss

**Figure 2:**
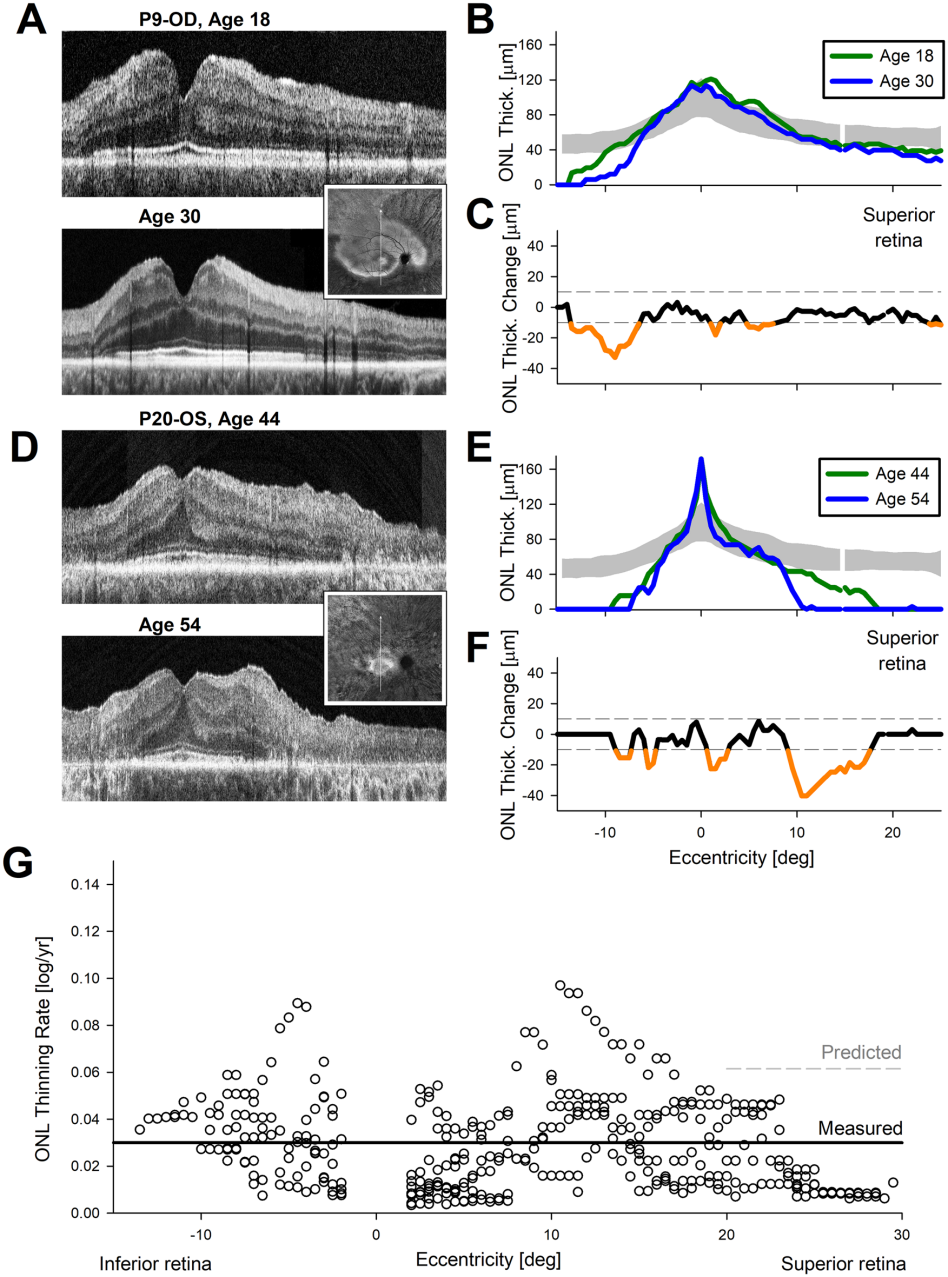
Slowly progressive thinning of photoreceptor outer nuclear layer (ONL) over a decade. (**A**, **D**) OCT scans along the vertical meridian crossing the fovea obtained at two ages separated by a decade or more in a younger (**A**) and older (**D**) subject. Insets, ultra-wide near-infrared autofluorescence imaging of the melanin-related pigments distinguishing darker retinal regions of demelanization and degeneration from the healthier regions with high signal levels. Arrows, location of the cross-sectional OCT scans shown. (**B**, **C**, **E**, **F**) ONL thickness at two ages (**B**, **E**) and their difference (**C**, **F**) as a function of retinal eccentricity. Dashed lines correspond to the expected variability, and orange sections of traces in panels (**C**) and (**F**) demarcate significant ONL changes. (**G**) ONL thinning rate across the cohort with long-term serial data as a function of eccentricity along the vertical meridian in retinal coordinates. The measured mean thinning rate (solid line) is smaller than the predicted rate (dashed) estimated from the mean rod vision loss.

In IRDs, the major driver of progressive vision loss is thought to be due to the degeneration of photoreceptor cells^35^. To define the rate of photoreceptor degeneration, we evaluated retinal structural changes from serial recordings performed over a long interval. Retinal cross-sectional scans with OCT were performed along the vertical meridian crossing the fovea to allow sampling of both pericentral and altitudinal defects previously described in Class B *RHO* patients^12^. Two representative patients illustrate the data collected over more than a decade. P9 had a region of retained retina surrounded by severe retinal disease (Fig. 2A, Inset). The region of retention extended to the superior macular and supero-temporal midperipheral areas. The OCT at age 18 showed thinning of photoreceptor outer nuclear layer (ONL) inferior to the fovea and there was asymmetric extension of retained photoreceptors into the superior retina (Fig. 2A, upper). Twelve years later, qualitatively OCT showed nearly no detectable changes (Fig. 2A, lower). Quantitation of the ONL thickness demonstrated a small but significant thinning occurring at around 9° inferior to the fovea (Fig. 2B,C). P20 represents a more severe stage of disease with only a central region of photoreceptors remaining (Fig. 2D, Inset). At age 44, there was asymmetry along the vertical meridian with ONL extending further into the superior retina compared to inferior retina (Fig. 2D, upper). Ten years later, qualitatively ONL extent had become more symmetric (Fig. 2D, lower). Quantitation showed significant thinning between 9 to 18° superior to the fovea with smaller changes occurring between 6 and 10° inferior to the fovea and in the superior parafovea (Fig. 2E,F).

In the cohort with serial OCT recordings over a long interval, there were 13 eyes of 8 patients with Class B *RHO*-adRP (Table S1). At first visit, average age was 44 (range 18–64) years. Last visit occurred on average 15 years later. Across all eyes, 49% of 1,482 sampled loci (excluding the cone-only fovea) had detectable ONL at the first visit, and the nominal progression rate was 0.017 log/year. The long interval between visits allowed differentiation of loci showing significant changes from loci showing changes within test–retest variability. The magnitude of change in 23% of retinal loci was smaller than the test–retest variability^36^. Remaining 26% of retinal loci with significant change showed a mean ONL thinning rate of 0.03 log/year (CI: 0.02 – 0.04 log/year; *p* = 0.0004, MEM, Fig. 2G). We next asked how the ONL thinning rate over the long term compared to the progression rate of visual sensitivity loss over the long term. Considering the majority of the extrafoveal retinal cells are rod photoreceptors, a simple model of structure–function relationship^37^ would predict the ONL thinning rate to be half of RSL progression rate. Thus, the RSL progression rate of 0.12 log/year (Fig. 1E–G) would predict an ONL thinning rate of 0.06 log/year (CI: 0.05 – 0.07 log/year, *p* = 0.0004, MEM, Fig. 2G, predicted). The measured ONL thinning rates (Fig. 2G, measured) however were slower than the rates expected, with no overlap of their CI.

In summary, long-term changes in visual function (Fig. 1) and retinal structure (Fig. 2) taken together support two key hypotheses for the natural history of Class B *RHO*-adRP. First, there are many retinal regions showing no evidence of structural and functional progression despite an ultra-long interval of observation over decades. Second, some retinal regions show progression however there appears to be an imbalance between structure and function with the latter progressing faster than the former.

### Detection of disease progression over a 2-year interval

Since most clinical trials evaluating promising therapeutic avenues for IRDs are planned for a short duration as compared to the protracted natural history of disease, we next evaluated detectability of disease progression over a 2-year interval in a larger cohort. Serial examination of rod and cone vision across the full visual field provided a macroscopic view (Fig. S1). P8 at age 25 is a representative result demonstrating mild loss of rod and cone sensitivity that is greater in the periphery and relatively less in the pericentral region (Fig. S1A). Two years later, qualitatively there are only minor changes with some improvements and some decrements consistent with what would be expected from test variability. Across 17 patients evaluated over a short term interval (median 2.0, range 2.0 to 2.9, mean 2.1 years; Table S2), the progression rates were not significantly different than zero (0.013 and 0.005 log/year, *p* = 0.61 and 0.59, for rod and cone function loss, respectively, Fig. S1B). There was no evidence for dependence on age or eccentricity (Fig. S1B; RSL: *p* = 0.9 and 0.7, CSL:*p* = 0.65 and 0.19 for Age and Eccentricity, respectively).

**Figure 3:**
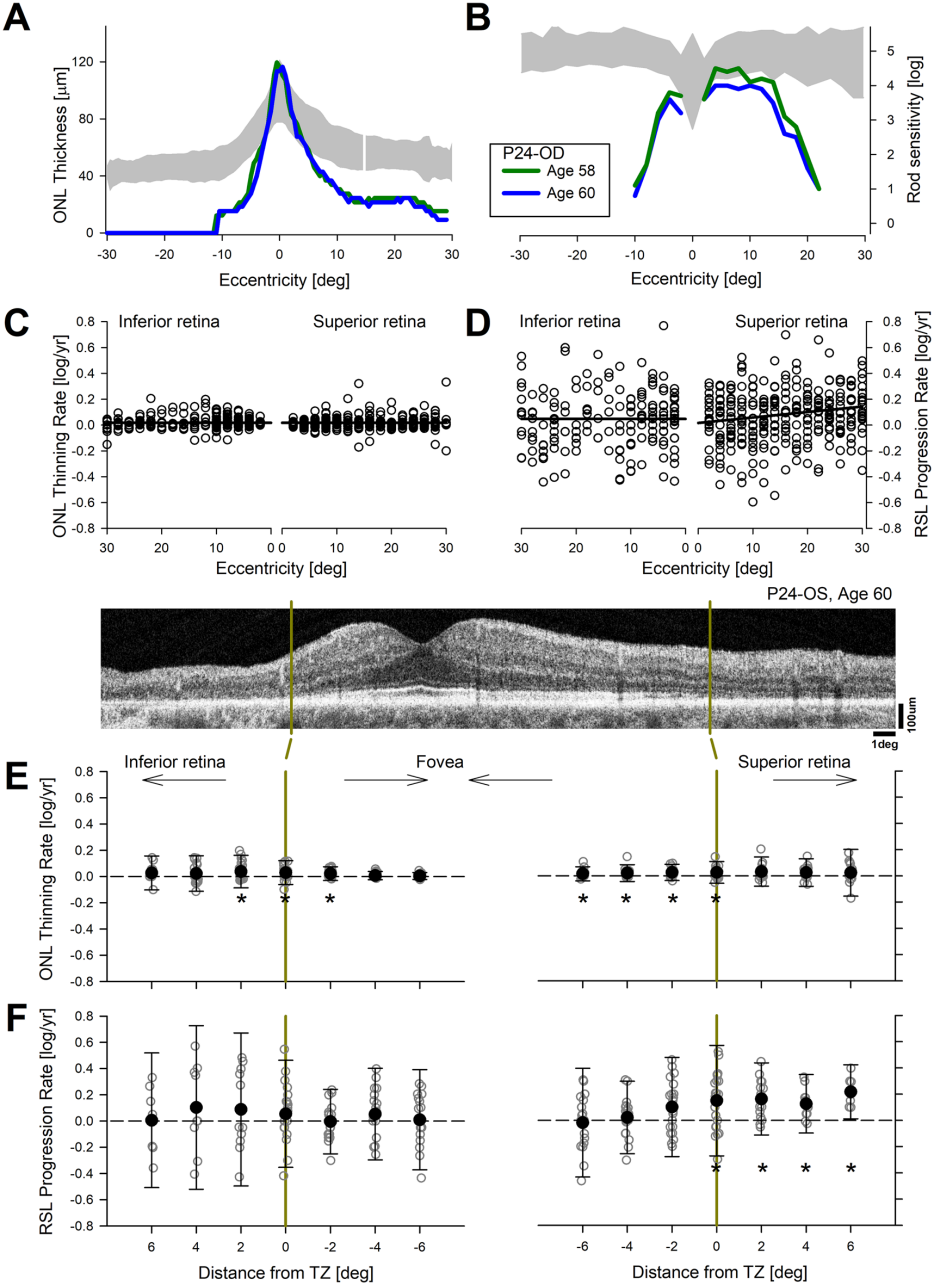
Disease progression over a short interval of 2 years. (**A**, **B**) ONL thickness (**A**) and RSL values (**B**) as a function of eccentricity along the vertical meridian in a representative patient obtained over 2 years shows little change that is distinguishable beyond variability. (**C**, **D**) Estimated ONL thinning (**C**) and RSL progression (**D**) rates as a function of eccentricity along the vertical meridian in inferior and superior retinas of a cohort of patients with serial data obtained over a 2 year interval. Linear regression results are shown with solid lines. (**E**, **F**) ONL thinning (**E**) and RSL progression (**F**) rates as a function of distance from transition zone (TZ) in the superior and inferior retinas. Error bars represent ± 1.96 SD to illustrate the population distribution. *Indicates locations with significantly different than zero mean rates. *Inset*, a representative OCT scan showing the superior and inferior TZ locations (yellow vertical lines).

To examine potential short-term changes at greater spatial detail, we also sampled central retinal structure and function along the vertical meridian within a 2-year interval (Fig. 3). The right eye of P24 at ages 58 and 60 was representative of the data acquired in this cohort of patients. ONL thickness was within the normal range centrally and became abnormally thin more eccentrically; superior retina had a larger expanse of retained photoreceptors compared to the inferior retina (Fig. 3A). There was little change in ONL thickness over 2 years. Colocalized rod-mediated function was mildly reduced centrally but severely reduced more eccentrically with a superior-inferior asymmetry (Fig. 3B). There were some reductions in sensitivity over the observation period but the changes were relatively small.

Across the whole cohort of eyes followed short-term (Table S2), disease progression estimated from the changes in ONL thickness showed mean rates of 0.02 log/year in both inferior and superior retina (Fig. 3C). Both rates were small although significantly different than zero (*p* < 0.001, MEM); there was no significant relation with eccentricity (*p* = 0.55 and 0.075 for inferior and superior retina, respectively, MEM). Co-localized rod-mediated function loss along the vertical meridian (Fig. 3D) was not significantly different than zero (estimate 0.05 log/year, *p* = 0.064, MEM). In the superior retina, mean RSL progression rate was 0.08 log/year (*p* < 0.0001, MEM) and showed a minor but significant slope with a tendency to have greater rates further eccentric to the fovea (eccentricity coefficient 0.004 (log/year)/deg, *p* = 0.0001). Overall RSL progression rate in the superior retina was larger than ONL thinning rate but variability was a significant impediment for detecting progression in the short term.

Previous work had shown detectable but small structural changes to subcellular structures of photoreceptors occurring at transition zones (TZs) between retinal disease and health in Class B *RHO*-adRP patients^13^. However, the retinal location of such TZs varies for each subject and eye and thus would be expected to be distributed at different eccentricities along the vertical meridian evaluated above. We asked whether more reliable rates of photoreceptor degeneration and rod vision loss could be obtained if data were shifted to align for each individual’s TZ. We plotted the rates as a function of distance to the TZ (Fig. 3E,F). There was statistically significant progression of ONL in both inferior and superior retina (Fig. 3E), however the magnitudes of the rates were small (0.02 and 0.03 log/year for inferior and superior retina, respectively, *p* < 0.0001, MEM) and significant thinning rates were located mostly at the TZ in the inferior retina and central to the TZ at the superior retina. In terms of rod function, mean progression rates were 0.05 and 0.1 log/year (*p* = 0.027 and < 0.0001 for inferior and superior retina, respectively, MEM). Significant function loss was located peripheral to the TZ at the superior retina. The mean rate of RSL progression at the superior retinal TZ was larger than the mean rate of ONL thinning similar to the results found with the long term data.

### An unexpected rod function deficit

Loss of vision in many IRDs are long thought to be spatially colocalized to loss of photoreceptors since the early days of retinal cross-sectional imaging^38,39^; over time, as photoreceptor cells are lost to progressive degeneration, vision would be expected to be lost proportionally. More recently, quantitative studies have shown rare exceptions to this “simple retinal degeneration” hypothesis: some congenital IRDs mostly within the diagnostic criteria of Leber congenital amaurosis demonstrate a dissociation of structure and function where there is a substantially greater visual function deficit than predicted from retinal structure^37,40^. However, RP in general, and *RHO*-adRP in specific, has not been thought to belong to this category of IRDs^3,10,41–44^. Our analyses thus far however appear to imply functional disease progression could be larger than expected from the underlying photoreceptor loss examined either over decades (Figs. 1,2) or over the short term (Fig. 3). Therefore, we decided to evaluate in detail the quantitative relationship between structure–function in Class B *RHO*-adRP patients.

**Figure 4:**
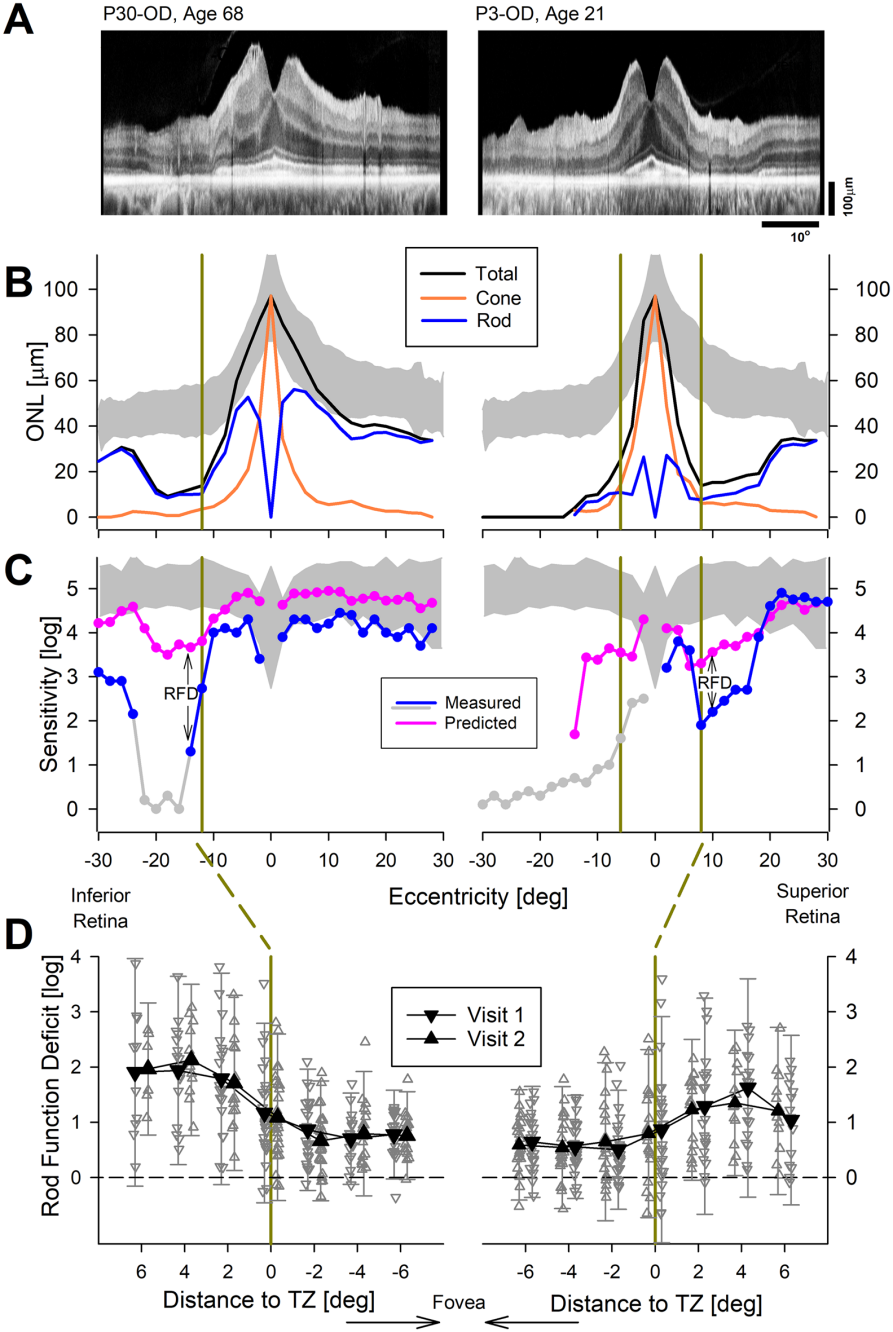
Evidence for a large rod function deficit. (**A**–**C**) Representative results along the vertical meridian from an older subject with a milder disease stage (left panels) and a younger subject with a more severe stage (right panels). OCT scans (**A**) are used to measure the total ONL thickness (**B**, black). A hybrid model provided estimates of cone (orange) and rod (blue) ONL thickness components. When measured rod sensitivity loss (RSL, C, blue) is compared to the predicted RSL (pink) there is a large rod function deficit (RFD) near transition zone (TZ). Gray symbols represent cone-mediated function; rod-mediated function at these locations are known to be below cone-mediated function. (**D**) Data from all eyes evaluated at two visits showing rod function deficit as a function of distance from the TZ in inferior and superior retinas. Smaller gray symbols represent individual data points, larger black symbols represent the means. Error bars represent ± 1.96 SD to illustrate the population distribution. Yellow vertical bars mark the TZs in individual eyes (**B**, **C**) and across the cohort (**D**).

First step in this endeavor was to make sure structure and function were matched for a given photoreceptor type: i.e. rod photoreceptor structure was matched to colocalized rod-mediated function. Rod function can be relatively easily differentiated from cone function (Figs. 1, 3 and refs^45,46^). However, by non-invasive imaging methods available to date, rod and cone nuclei within the ONL are not directly distinguishable by their backscatter characteristics. Therefore, we developed a hybrid method to differentiate the rod and cone ONL components in OCT images (Supplementary Methods and Fig. S3). Results from two representative patients serve to illustrate the data. An OCT scan from P30 with a mild disease stage at age 68 showed a photoreceptor defect in the inferior pericentral retina (Fig. 4A, left). Overall ONL thickness (Fig. 4B, black) was normal or near normal throughout the region except for the photoreceptor defect. The hybrid model provided an estimate of the rod (Fig. 4B, blue) and cone (Fig. 4B, orange) photoreceptor components of the ONL. Predicted rod sensitivity based on rod components of the ONL thickness was near normal except for the inferior paracentral defect (Fig. 4C, pink). However, measured rod sensitivity values were lower than predicted values throughout the retina (Fig. 4C, blue). The difference between the measured rod function and predicted rod function was defined as the rod function deficit (Fig. 4C, RFD), which had the largest magnitude in the vicinity of the photoreceptor defect. P3 at age 21 illustrates a more severe stage of disease with substantial loss of photoreceptors in the inferior retina. Superior to the fovea, there was a pericentral loss of photoreceptors (Fig. 4A, right). RFD was greatest across the superior pericentral defect (Fig. 4C, right). In addition, there was an implied RFD inferior to the fovea where rod function was so deficient not to be able to be detected.

The magnitude of the RFD was estimated across the whole cohort of eyes within the neighborhood of the superior and inferior retinal TZ (Fig. 4D). Measurements were performed on two visits (Table S3). At the first visit, central to the TZ, average RFD was 0.8 log in the inferior retina, and 0.6 log in the superior retina. The RFD grew to 1.2 and 0.9 log at the TZ for inferior and superior retina, respectively. Peripheral to the TZ, RFD grew further reaching up to an average value of 1.9 and 1.6 log units, for inferior and superior retina, respectively (Fig. 4D). At the second visit performed 2.1 years later, the RFD magnitudes central to the TZ were 0.7 log in the inferior retina, and 0.6 log in the superior retina; peripheral to the TZ, RFDs extended up to 2.1 and 1.4 log for inferior and superior retina, respectively. Existence of a large and invariant RFD (well beyond the expected variability of rod function measures) implies that a successful treatment directed at rod photoreceptors can potentially result in large improvements in night vision.

### Progression and transition zones in a canine model

**Figure 5:**
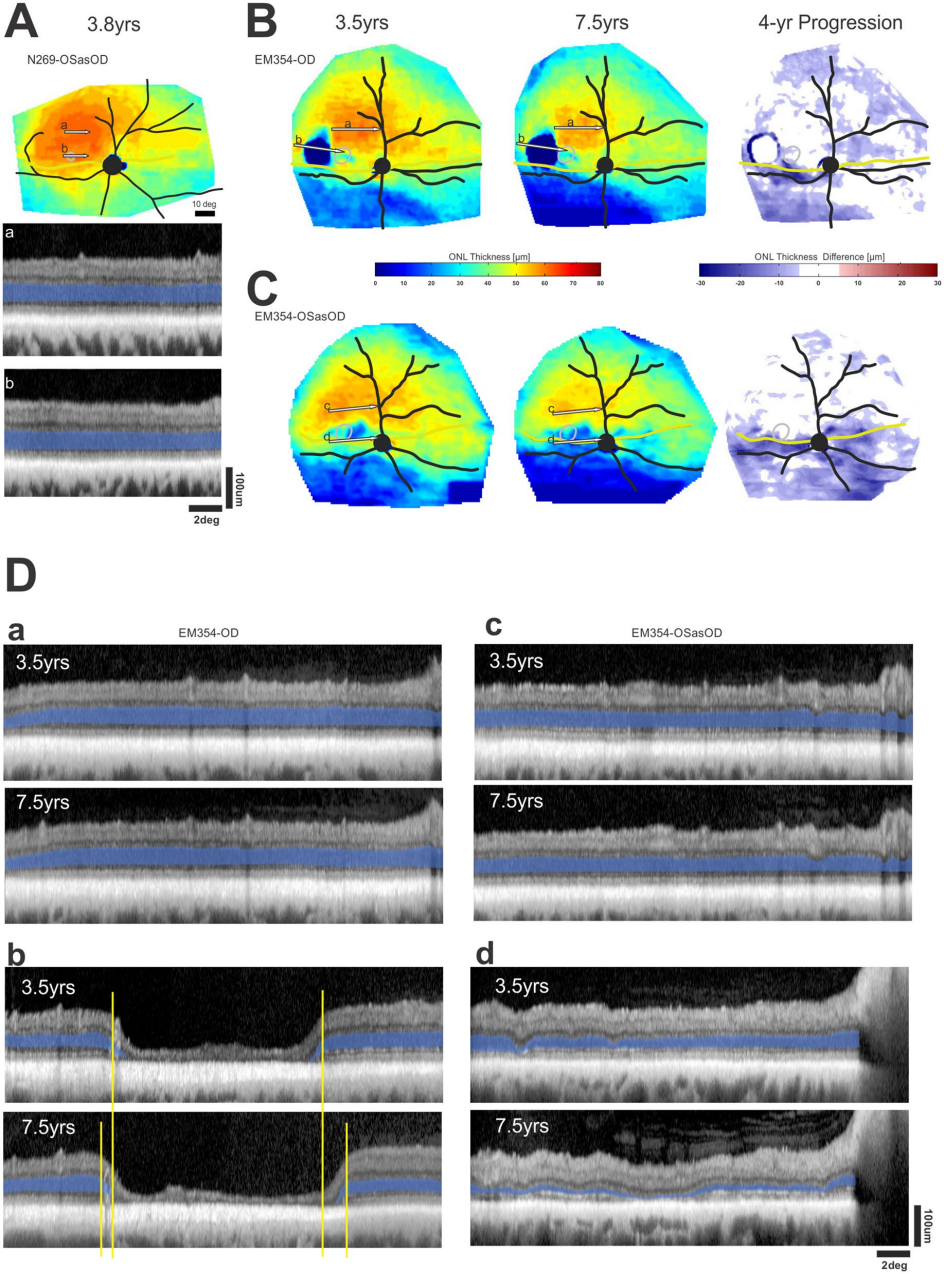
The natural history of photoreceptor degeneration in *RHO*-mutant canine eyes. (**A**) Pseudocolor ONL thickness map in a WT dog (upper) and representative OCT scans from superior-temporal (**a**) and temporal (**b**) retinal regions (lower). (**B**, **C**) Pseudocolor ONL thickness maps (left and middle) in two eyes of a dog at ages 3.5 and 7.5 years. The dog was kept in regular cyclic kennel light in order to glean the natural history of RHO disease. Difference maps (right) show the spatial distribution of change in ONL thickness occurring over the 4 year interval. White represents change being within ± 6 µm, red thickening and blue thinning. (**D**) OCT scans representing superior-temporal regions of little progression (**a**, **c**), and temporal region of substantial progression (**b**, **d**) over the 4 year serial followup. Yellow lines (**D**, **b**) show the expansion of the transition zone.

Some features of retinal disease of patients with Class B *RHO*-adRP are modeled well in a naturally occurring dominant retinopathy due to a T4R mutation in the canine *RHO* gene^5,47,48^. To better understand the details of the canine natural history, serial imaging was performed in RHO^T4R/+^ eyes at 3.5 and 7.5 years of age (Fig. 5). The corresponding human ages would be 21 and 46 years by allometric scaling^9^, or 50 and 62 years by molecular aging^49^. Initially there were large regions of superior retina which showed no evidence of photoreceptor degeneration, there were smaller central regions and a swath of infero-temporal retina with loss of photoreceptors (Fig. 5B,C,D). Serial imaging 4 years later (corresponding to a 25 or 12 year interval for humans^9,49^) showed many central regions with near normal ONL thickness and little progressive retinal degeneration(Fig. 5Ba,Da,Cc,Dc). Infero-temporal region showed further photoreceptor loss (Fig. 5B,C). Central region of atrophy had centrifugally expanded in one eye (Fig. 5Bb,Db), and showed definite loss of photoreceptors in the other eye (Fig. 5Cd,Dd). The progressive movement of the TZ over 4 years was 0.2 to 0.4 mm corresponding to 0.23 to 0.45 deg per canine year (Fig. 5Db). To assess the morphology of photoreceptors located near the TZ, H&E stained retinal cryosections whose location and orientation was identified on the ONL thickness map were examined in a different dog (Fig. S2). A gradual increase in thickness of the ONL was seen over distances ranging from ~ 100–400 µm. Within this TZ, rod photoreceptors showed inner segments that were absent to shortened, and outer segments material that was absent to limited (Fig. S2).

## Discussion

How does this research into *RHO*-adRP fit in the larger scheme? Among the approximately 7,000 rare diseases, it is estimated that 80% are genetic in origin^50^. Within the genetic eye disease category are those that affect the retinal photoreceptors and lead to blindness. Earlier eras of disease discovery led to naming of these progressive retinal blinding disorders by phenotype; and all Mendelian genetic types were identified. In more recent times mechanisms of the diseases have become better understood and causative genes were elucidated^4^. Now, there is increased focus on therapeutics of previously incurable retinal blindness. Armed with all of this knowledge and the desire to begin treatments, specifically gene-based therapies, there is a need to determine ways to quantify the abnormal vision and decide whether the therapy will be not only safe but efficacious. Preservation and/or improvement of photoreceptor function are the key therapeutic efficacy goals.

To date, all human clinical trials of inherited retinal degenerations using gene-based treatments and one approved gene therapy product have been for autosomal recessive and X-linked forms where loss of gene function is the basic pathology. For dominant forms of retinal disease, on the other hand, translation from pre-clinical successes to the clinic has been more challenging^5,6^. Given recent proof-of-concept efficacy in the canine model of *RHO*-adRP^5^, there is strong reason to move toward a clinical trial and determine an outcome measure based on the mutant rod photoreceptors. The current study identifies a previously unrecognized large functional deficit in retained rod photoreceptors of patients with a common form of dominant IRD and thus provides a key therapeutic goal that can likely be achieved with efficacious gene-based therapies.

### Natural history of rod photoreceptor dysfunction in human IRDs

Rods are the most numerous photoreceptors in the human retina^51^ and they are severely affected in most IRDs. Progression rates of rod-mediated dysfunction, however, are very rarely assayed in the clinic. Instead, for a number of practical reasons, disease progression in patients with IRDs has mainly been quantified using cone-only measures of function. In large studies which followed patients long-term, the rate of progression of the cone electroretinogram (ERG) amplitude was reported to be 8.7%/year (0.04 log/year) for *RHO*-adRP and in the range of 10 to 17.1%/year (0.05 to 0.08 log/year) for all RP^34,52–54^. Our estimate of perceptual cone sensitivity progression in the current work in Class B *RHO*-adRP evaluated serially over two decades averaged across the functional visual field was 0.04 log/year and was thus comparable to the previous work based on full-field ERGs. Importantly, however, our use of spatial sampling of cone vision across the visual field demonstrated the existence of two populations of photoreceptors. The majority of retinal locations were showing no detectable progression despite our two-decade-long observation interval. In contrast, there were some retinal locations, mostly near health-disease transitions, that showed a substantially faster progression of cone function loss at 0.07 log/year.

There is no comparative literature on longitudinal progression of rod function loss in Class B *RHO*-adRP. Cross-sectional studies have shown that large intra-retinal differences in severity of rod dysfunction^3,8,10–12,55^ implying a natural history of spatio-temporal differences in disease progression. The current work provides direct support for this hypothesis by demonstrating the existence of large retinal regions with stable rod function over two decades in a primary rod photoreceptor disease. Near regions of stability, however, were also areas showing progression of rod function at a rate of 0.123 log/year (24.7%/year). The rate of the progressive loss of rod function in Class B *RHO*-adRP was similar to the rate estimated for IRDs associated with *ABCA4* mutations^29^, but appeared to be slower than the estimates for IRDs associated with *USH2A* or *RPGR* ^33,56^. Progression of rod ERG amplitude reduction^54^ appears to be substantially slower than the perceptual rod dysfunction. Our longitudinal rod and cone function studies over two decades taken together with previous studies imply that interventions intended to preserve vision would need to reliably predict retinal loci that are destined for progression in order to evaluate treatment efficacy. Even then, clinical trial durations would have to be longer than 5 years to be able to demonstrate a positive change to the slow natural history of vision loss.

### Natural history of retinal structure

The gold standard for understanding retinal structural defects in human IRDs has been microscopic evaluation of postmortem retinal tissue. Rare eye donors with Class B *RHO*-ADRP have shown a wide spectrum of retinal disease severity ranging from near normal photoreceptors with near normal outer segments, to partial loss of photoreceptors with shortened and disorganized outer segments, to complete loss of photoreceptors^8,35,57–62^. With the advent of OCT^63^ examination of photoreceptors in living IRD eyes became possible^38,39,64–66^ and led to micron scale quantitative studies of subcellular components such as the ONL thickness^67^ and inner and outer segment layers^68^. The current work showed that some retinal regions had a slow but detectable rate of degeneration of 0.03 log/year (or 7%/year) over a longitudinal interval of 15 years. We are not aware of any studies in Class B *RHO*-adRP quantifying ONL thinning, and aware of only one other study in any IRD^69^ which showed an ONL thinning rate of 0.04 log/year over a longitudinal observation period of 5 years. Similar to the implications of slow progression of vision loss, we conclude that retinal degeneration, as defined by loss of photoreceptor nuclei, is difficult to detect in Class B *RHO*-adRP. Required are reliable predictors of retinal loci destined for future degeneration and relatively long observation periods.

### Vulnerable retina at the transition zones

Disease progression in IRDs occurs in a complex spatio-temporal pattern^3,12,30,48,70–74^. One approach in quantifying progression is to follow the boundaries of TZ between disease and health over time in order to define either the expansion of retinal area of disease or constriction of area of health. Movement of TZ over time is likely driven by non-autonomous cell death mechanisms acting within or across photoreceptor types^75–84^. Importantly, definition of the TZ varies across investigations and can include some aspect of visual function^11,52,85–87^, RPE atrophy or demelanization^13,73,88–90^, extent of normal ONL thickness^71,87,91^, or the extent of the IS/OS signal detectability on OCT which demarcates severe OS abnormality^12,13,89,92–95^. In a previous investigation, we showed that spatial progression of TZ as defined by the detectability of the IS/OS signal was greatest in the superior retina over a 2-year interval in Class B *RHO*-adRP^13^. Therefore, we used the same definition of TZ in the current study to evaluate the temporal progression of key disease parameters at fixed retinal locations in the region immediately central to, centered on, and immediately peripheral to the TZ. Over a 2-year observation period, there was little detectable ONL thinning at these vulnerable retinal loci (Fig. 3E). In the superior retina, significant progression of rod sensitivity loss was detectable (Fig. 3F) exactly where constriction rate of the TZ boundary was previously shown to have the largest progression rate^13^. Thus, our results continue to support the superior retinal TZ as the most vulnerable retinal locus in Class B *RHO*-adRP where consequences of disease progression may be most detectable in the shortest interval.

### Quantitative relation between structure and function

Understanding the relationship between co-localized retinal structure and visual function is key for determining vision improvement potential with treatments in IRDs^40^. Studies of rod-mediated function and retinal structure in extra-foveal regions dominated by rods have been mostly qualitative^10,12,38,39,64,96–100^. Rare quantitative studies made simplifying assumptions and limited analyses to foveal and rod-hot-spot regions of the retina to circumvent the complication resulting from the mixture of rod and cone nuclei in the ONL^37,41–43,101,102^. The hybrid model developed for the current study allowed relaxation of some of the assumptions and quantitatively examined retinal regions with comparable rod and cone densities such as the perifoveal region where TZs are often located in many IRDs. The result was the discovery of a large RFD which was especially prominent immediately peripheral to TZ areas. In retrospect this finding is maybe not surprising. Peripheral to TZ, retinal function is substantially reduced, ONL thickness is relatively retained while OS are very abnormal in many IRDs^12,87,93,103,104^. Structural abnormalities of the rod OS beyond that expected from loss of neighboring rod cells and functional abnormalities of the rod system beyond that expected from reduced OS volume could combine to contribute to the RFD. Mechanisms underlying cellular pathology could include misfolding and mislocalization of phototransduction proteins, downregulation of non-phototransduction proteins, ER stress or a combination of these effects^7,11,14,15^. Whether therapies can improve rod OS abnormalities in Class B *RHO*-adRP remains unknown, but some animal studies suggest that the potential exists^105,106^.

### Potential consequences of efficacious therapies

Arresting or slowing the spatial movement of the TZ in IRDs has been considered a reasonable surrogate endpoint for evaluating the efficacy of interventions^13,94^. Our results support this approach in Class B *RHO*-adRP, especially in the superior retina. However, it is important to note that progression rates are slow and detection of significant change may take longer periods of observation. Improvement of rod function is a new goal that is now supported by data instead of speculation. The largest deficits of rod function, and thus largest potential for improvement was in retinal regions with evidence of rod nuclei lacking outer segments. Whether these severely diseased rods can regenerate outer segments remains to be determined. But it is important to note that wider retinal regions with milder disease stages also showed a RFD, albeit with a smaller magnitude. Incremental improvement of rod OS structure and function within these healthier regions may be a more realistic goal of early therapies.

## Methods

### Subjects

The study population consisted of patients with *RHO* gene mutations and Class B phenotype (n = 30); subsets of patients were re-evaluated with functional or structural methods over long term (~ 20 years) or short term (~ 2 years) intervals in order to better understand the natural history of disease (Tables S1–S3). A complete eye examination was performed in all subjects, including best-corrected ETDRS visual acuity and kinetic visual fields. Some aspects of all patients have been published previously^8–13^ but all data and analyses shown in the current work are novel. Patient numbers in the current work correspond to patient numbers published recently^13^ to allow comparison. In the subset of patients evaluated over the long term, a large percentage (87%) reported taking supplements of vitamin A^53^ for varying periods of time when they were monitored. Lesser numbers (68%) reported taking lutein supplements. None of the patients took the excess nutrients for the duration of the time they were monitored; most patients took the supplements anticipating improvement and after about 2–5 years stopped taking them when expectations were not met or when side effects occurred. Procedures followed the Declaration of Helsinki, and the study was approved by the Institutional Review Board (IRB) of the University of Pennsylvania. Informed consent, assent, and parental permission were obtained, and the work was HIPAA-compliant.

### Measures of rod and cone function

Two-color dark-adapted perimetry was used to measure rod-mediated function across the visual field^8,45,107^. Mediation of the 500 nm (blue–green) stimulus (1.7° diameter; 200 ms duration) sensitivity by rod photoreceptors was determined by comparison of sensitivities with a 650 nm (red) stimulus and taking advantage of the spectral sensitivity differences between rods and cones. Light-adapted perimetry with a 600 nm (orange) stimulus was used to measure cone-mediated function. All tested eyes had foveal fixation, and sensitivity measurements were sampled at 12° intervals across the full visual field (120° wide and 84° high) or at 2° intervals along the vertical meridian (60° in extent) in the central retina. All measures of rod and cone function are provided as loss from mean normal sensitivity at the location evaluated, considering rod and cone sensitivities vary across the normal visual field. Progression rates are provided in log_10_/year which can be converted to dB/year by multiplying with a factor of 10. In order to minimize floor effects in progression rate estimates, only locations with mild to moderate sensitivity loss (up to 3 log units of loss for rods, and up to 1.5 log units of loss for cones) at the first visit were considered. We assumed test–retest variability for rod and cone function in Class B *RHO*-adRP was comparable to our recent report in a different IRD examined with the same methods^33^.

### Retinal imaging

A confocal scanning laser ophthalmoscope (Spectralis HRA, Heidelberg Engineering, Heidelberg, Germany) was used to obtain near-infrared excited reduced-illuminance autofluorescence imaging (NIR-RAFI) with a laser output setting of 100% and detector sensitivity of 105%^13,30,88,108^. Both 30° and 55° lenses were used to obtain wide field coverage in a room with dimmed ambient lights. All images were acquired with the high speed mode. Wide field image montage was assembled by manually specifying corresponding retinal landmark pairs in overlapping segments using custom-written software (MATLAB 6.5).

Optical coherence tomography (OCT) was performed mainly with a spectral-domain (SD) OCT system (RTVue-100, Optovue Inc., Fremont, CA). Earlier visits of some patients were obtained with time domain OCT (OCT1 and OCT3; Carl Zeiss Meditec, Dublin, CA). Our recording and analysis techniques have been published^13,37^. In brief, the OCT protocol included 4.5 or 9 mm line scans along horizontal and vertical meridians crossing the fovea and extending to 9 mm into the periphery. Each scan was repeated 3 or more times. Post-acquisition processing of OCT data was performed with custom programs (MATLAB, MathWorks, Natick, MA). Scans were aligned by straightening the major RPE reflection and overlapping scans digitally merged to cover up to 30° in each direction. Repeated TD-OCT scans were aligned and averaged to get scans with higher signal-to-noise ratio. Quantitation of retinal layers was performed manually as a function of eccentricity every 0.5° using published methods. The hyposcattering ONL layer was defined between the hyperscattering outer plexiform layer and the hyperscattering outer limiting membrane and includes the anatomical layers of both ONL and Henle fiber layer. TZ was defined as the location where the IS/OS band becomes indistinguishable from the RPE along the vertical meridian.

### Statistics and progression rates

All rates of progression are presented in log per year assuming the natural history of disease is well described by a delayed exponential^78,80^. Positive values of the rates represent either loss of function or loss of ONL thickness over time. Estimations of means, 95% confidence intervals (CI) and relationships of progression of ONL thickness, and rod/cone sensitivity losses versus eccentricity, distance from transition zone and age were studied using a mixed-effects model (MEM) with Subject and Eye(nested) random effects, restricted maximum likelihood estimation and the Satterhwaite’s approximation for denominator degrees of freedom. Multiple comparisons were evaluated using Bonferroni corrections. Computations used the lme4 (ver. 1.1–21)^109^ and lmerTest (ver. 3.1–1)^110^ packages from R statistical software (ver. 3.6.1, 2019–07-05 3.4.4)^111^.

### Canine studies

Dogs were bred and maintained at the University of Pennsylvania Retinal Disease Studies Facility (RDSF) and housed under standard kennel cyclic (12 h ON, 12 h OFF) white light illumination (175–350 lx at the level of the “standard” dog eye). These included two RHO^T4R/+^ and one WT dog. The studies were carried out in strict accordance with the recommendations in the NIH Guide for the Care and Use of Laboratory Animals and the US Department of Agriculture Animal Welfare Act and Animal Welfare Regulations and complied with the Association for Research in Vision and Ophthalmology Statement for the Use of Animals in Ophthalmic and Vision Research. The protocols were approved by the Institutional Animal Care and Use Committee of the University of Pennsylvania. Details of imaging and histology have been published^5^.

## Supplementary information

Supplementary information
