# Supplementary material for: Rod function deficit in retained photoreceptors of patients with class B Rhodopsin mutations
Source: Sci Rep. 2020 Jul 28;10:12552. doi: 10.1038/s41598-020-69456-3 (PMC7387454; doi:10.1038/s41598-020-69456-3)
Supplement: Supplementary file 1 — Supplementary information [file 41598_2020_69456_MOESM1_ESM.pdf]

Supplementary Information for

# Rod Function Deficit in Retained Photoreceptors of Patients with Class B Rhodopsin Mutations

Artur V. Cideciyan, Samuel G. Jacobson, Alejandro J. Roman, Alexander Sumaroka, Vivian Wu, Jason Charng, Brianna Lisi, Malgorzata Swider, Gustavo D. Aguirre, William A. Beltran

**Supplementary Methods**

**Modeling the relationship between structure and function.**

Light received from the environment is focused by the cornea and the lens onto outer retinal photoreceptors. Phototransduction of light to a sensory signal starts in ciliary structures of photoreceptors called outer segments which contain high density of photolabile opsin molecules. In inherited retinal diseases (IRDs) that primarily affect the photoreceptors, abnormal outer segments result in a reduction of opsin molecules, and a consequent reduction in the probability of photons absorbed, and a decrease in the light sensitivity of the eye. Quantitative analysis of the relationship between opsins and light sensitivity is difficult in patients even with the simplifying assumption that photoreceptor synapse and post-receptoral circuitry is functioning normally. With early attempts using fundus reflectometry to estimate rod opsin pigment density, it was found that IRDs may differ substantially. Some patients showed a reduction of absolute rod-mediated sensitivity that was proportional to the reduction in rhodopsin density and thus were considered to be “consistent with the quantum catch hypothesis”; whereas other patients showed much larger losses of sensitivity than expected from opsin levels (1-4). Similar analyses could also be extended to cone photoreceptors and cone vision (5-7).

Earlier studies however could not differentiate between degeneration of photoreceptor cells (evidenced by the loss of their nuclei) and shortening of their outer segments. With the advent of optical coherence tomography (OCT), degeneration of photoreceptors could be measured directly by non-invasive cross-sectional imaging (8,9). Taking advantage of OCT technology, we previously developed a simple model of structure-function relationship and estimated the potential for vision improvement in an IRD due to a visual cycle defect (10). Successful gene augmentation therapy was able to demonstrate realization of this potential locally in treated retinal regions in human patients (11). Our model used the quantum catch hypothesis and assumed that vision loss at a given retinal location would be proportional to the product of the number of outer segments and their volume. Using a series of simplifying assumptions, we concluded that to a first approximation, loss of light sensitivity (in linear units) would be expected to be proportional to the square of thinning of the ONL where all photoreceptor nuclei are located (10).

Current imaging methods do not distinguish between rod and cone photoreceptor nuclei within the ONL, but sensitivity of the two photoreceptor systems can be distinguished. Therefore, we previously limited our analyses to the fovea where all photoreceptors are expected to be cones and can be directly compared to cone function, and to the “rod ring” (12) where rods dominate the photoreceptor population and can be compared to rod function (10,13-16). Important for the current work however was the evaluation of structure-function relationship at parafoveal regions where rod and cone densities can be comparable. We first used data from special cohorts of patients to estimate the expected cone component of the normal ONL. Six RP patients were selected for having no rod function across the retina and having normal cone function in the central retina (17,18). A representative result is shown (Fig. S3A). ONL thickness measures at retinal locations with normal cone function in RP patients were collected along the vertical meridian (Fig. S3C, light gray traces). In addition, 3 patients with

*CEP290*-LCA were included (Fig. S3B) and ONL thicknesses collected along the vertical profile (Fig. S3C, dark-gray traces). The average ONL thickness in these patients was assumed to represent normal cone component (Fig. S3C, orange trace) based on previous work (18,19). The estimated cone and rod ONL components are highlighted on a representative normal OCT based on the expectation from histology that the cone nuclei are always located more distal than rod nuclei (Fig. S3D). Thickness estimates of rod and cone ONL components demonstrate the cross-over at the parafovea where the retina changes from all-cone fovea to a mostly-rod periphery (Fig. S3E). The proportion of the ONL corresponding to the cones as estimated from imaging corresponds closely to the estimate from histology at the paraffoveal location (20, Fig. S3F). Of note, our measures of ONL include the histologically defined layers of Henle fiber layer (HFL) and photoreceptor nuclear layer. For the current work, we assume that HFL has rod and cone components proportional to the local rod and cone densities, and HFL components thin proportionally to the loss of rod and cone nuclei.

A hybrid model was developed to predict rod function deficit (RFD) at each retinal location in individual *RHO*-adRP eyes. We first measured dark-adapted absolute sensitivity to two colors (500 and 650 nm) and used the difference to determine whether the 500 nm sensitivity was mediated by rods (21,22). For all retinal locations where 500 nm blue sensitivity was mediated by rods, rod sensitivity loss was defined as:

$$P_{RSL}\ (x,y) = N_{RS}\ (x,y) - P_{RS}\ (x,y) \qquad (1)$$

where (x,y) are the retinal location coordinates in degrees of visual angle from fixation, $P_{RSL}$ and $P_{RS}$ are patient specific estimates in log units for rod sensitivity loss and rod sensitivity, respectively, and $N_{RS}$ is the mean normal rod sensitivity also in log units. Sensitivity is the inverse of threshold. Similarly light-adapted (white 10 $cd.m^{-2}$) increment sensitivities were measured with orange (600 nm) stimuli to estimate cone sensitivity loss at each location:

$$P_{CSL}\ (x,y) = N_{CS}\ (x,y) - P_{CS}\ (x,y) \qquad (2)$$

where $P_{CSL}$ and $P_{CS}$ are patient specific estimates in log units for cone sensitivity loss and cone sensitivity, respectively, and $N_{CS}$ is the mean normal cone sensitivity also in log units. Next, we used localized cone sensitivity losses to estimate local cone ONL thickness as:

$$P_{ONL\text{-}C}\ (x,y) = N_{ONL\text{-}C}\ (x,y) * 10\text{^}\ \{\ 0.5*P_{CSL}\ (x,y)\ \} \qquad (3)$$

where $P_{ONL\text{-}C}$ is the patient specific cone ONL thickness and $N_{ONL\text{-}C}$ is the mean normal cone ONL thickness. The factor 0.5 originates from the simple quantum catch model we previously developed (10) which assumes that outer segment length is reduced in proportion to loss of photoreceptors. Rod component of the ONL thickness is defined as:

$$P_{ONL\text{-}R}\ (x,y) = P_{ONL\text{-}T}\ (x,y) - P_{ONL\text{-}C}\ (x,y) \qquad (4)$$

where $P_{ONL\text{-}T}$ is the total ONL thickness measures, and $P_{ONL\text{-}R}$ is the rod ONL thickness estimated. Finally, the rod function deficit is defined as the difference between the predicted and measured rod sensitivity losses:

$$P_{RFD}\ (x,y) = P_{RSL}\ (x,y) – 2*\ \log\ \{\ P_{ONL\text{-}R}(x,y)\ /\ N_{ONL\text{-}R}(x,y)\ \} \qquad (5)$$

where $P_{RFD}$ is the estimated rod function deficit, $P_{RSL}$ is the measured rod sensitivity loss. The factor 2 originates from the simple quantum catch model (10).

It is important to note that the length of rod and cone outer segments can be estimated in normal or near-normal retinal regions (23) and explicit incorporation of the outer segment length into estimating sensitivity has been previously used (15,24-26). However, in degenerate retinas, with shortening of the outer segments it is often not possible to distinguish between rod and cone outer segments (except at the fovea), or even distinguish any outer segments at all. Therefore, our model used the implicit inclusion of outer segment length being proportional to remaining fraction of photoreceptors (10). This approach provides a conservative bound to the potential for rod sensitivity improvement. In the best-case scenario, improvements may be greater than implied with our RFD estimate if rod outer segments in regions of partial photoreceptor degeneration can grow to normal length.

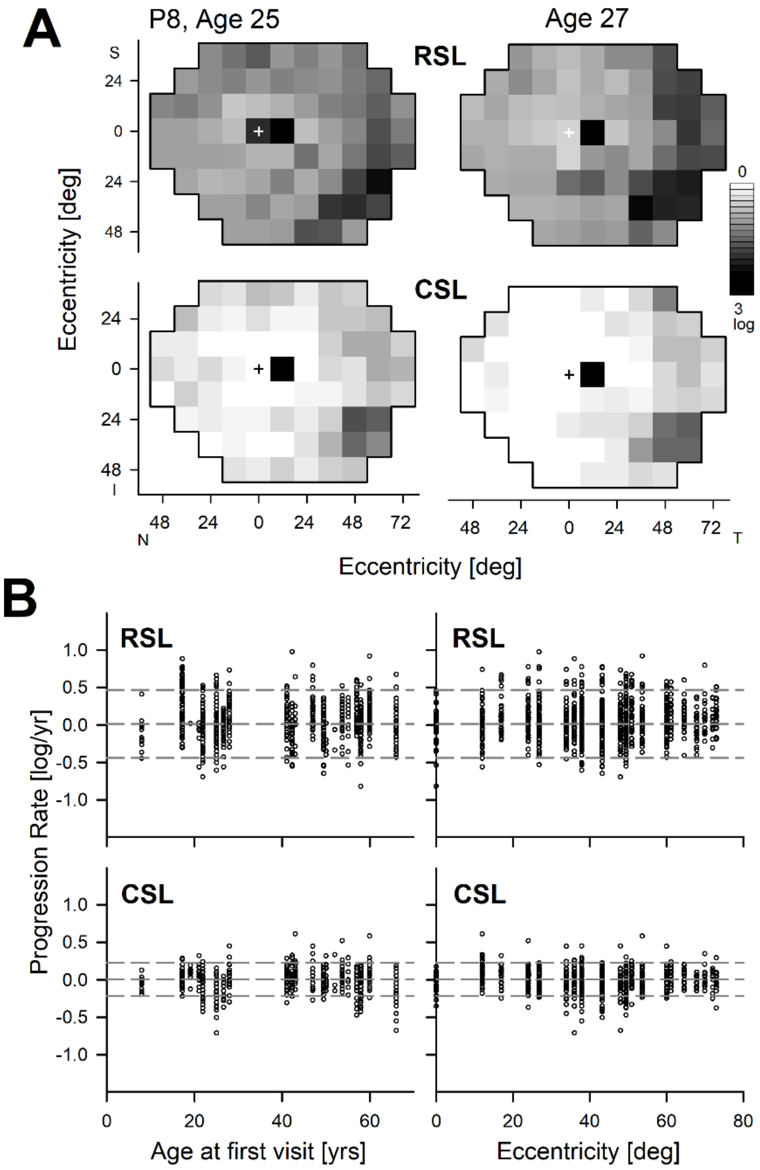


**Fig. S1.** Retina-wide measures of rod and cone sensitivity loss (RSL and CSL) in a two-year interval. (**A**) RSL and CSL results mapped on grayscale from a representative patient show a region of retained function in the central and infero-nasal regions but no significant change over the two-year interval. (**B**) Progression rates of RSL (upper) and CSL (lower). There are no significant effects of age (left) or eccentricity (right). Dashed lines represent mean±1.96Std and cover ~95% of the population distribution.

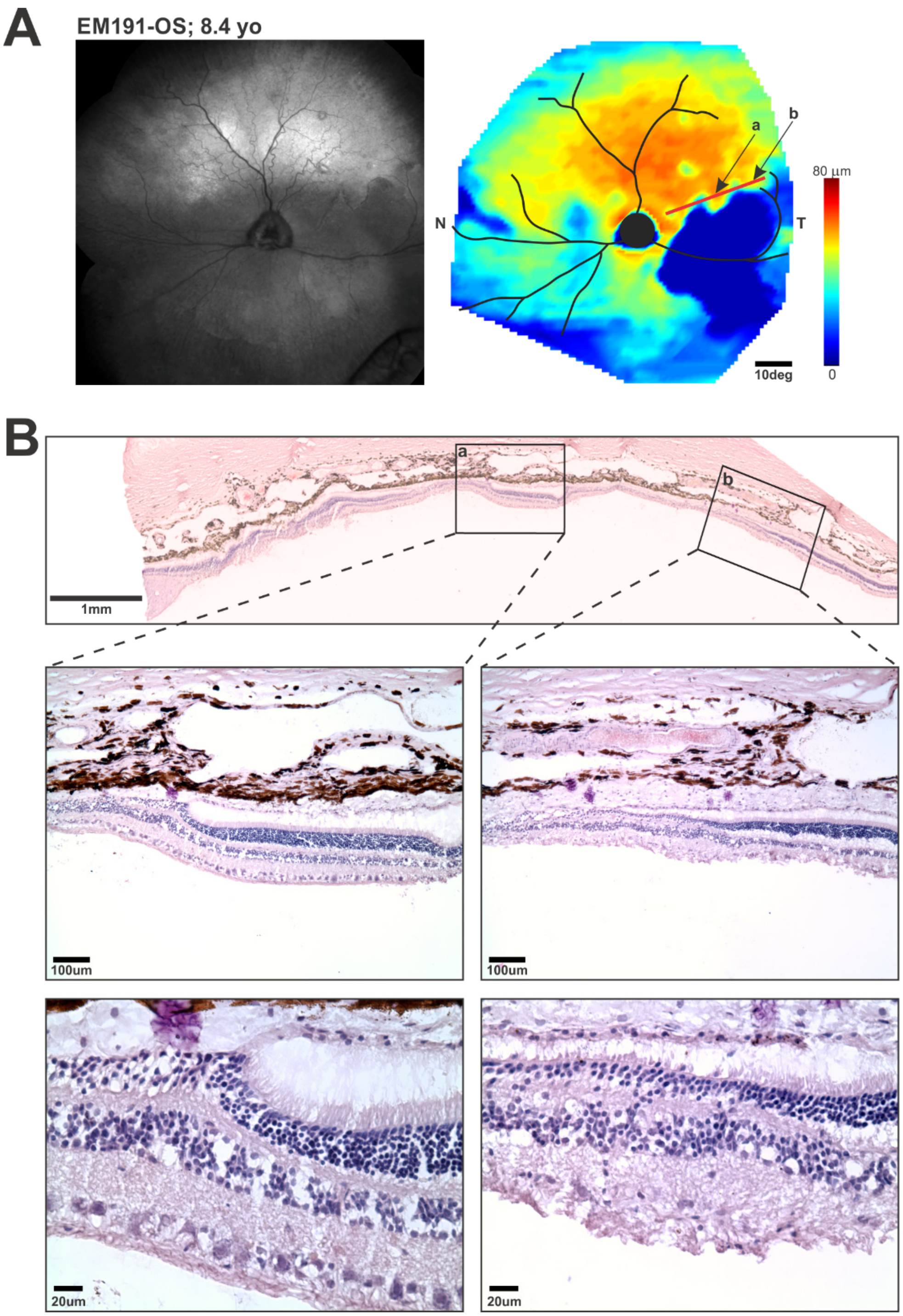


**Fig. S2.** Histology of the transition zone in the naturally occurring canine model of Class B *RHO* disease. (**A**) Infrared reflectance image and pseudocolor ONL thickness map showing the location of the section (red line). (**B**) Representative H&E stained retinal cryosection adjacent to the region of atrophy. Higher magnification shown below.

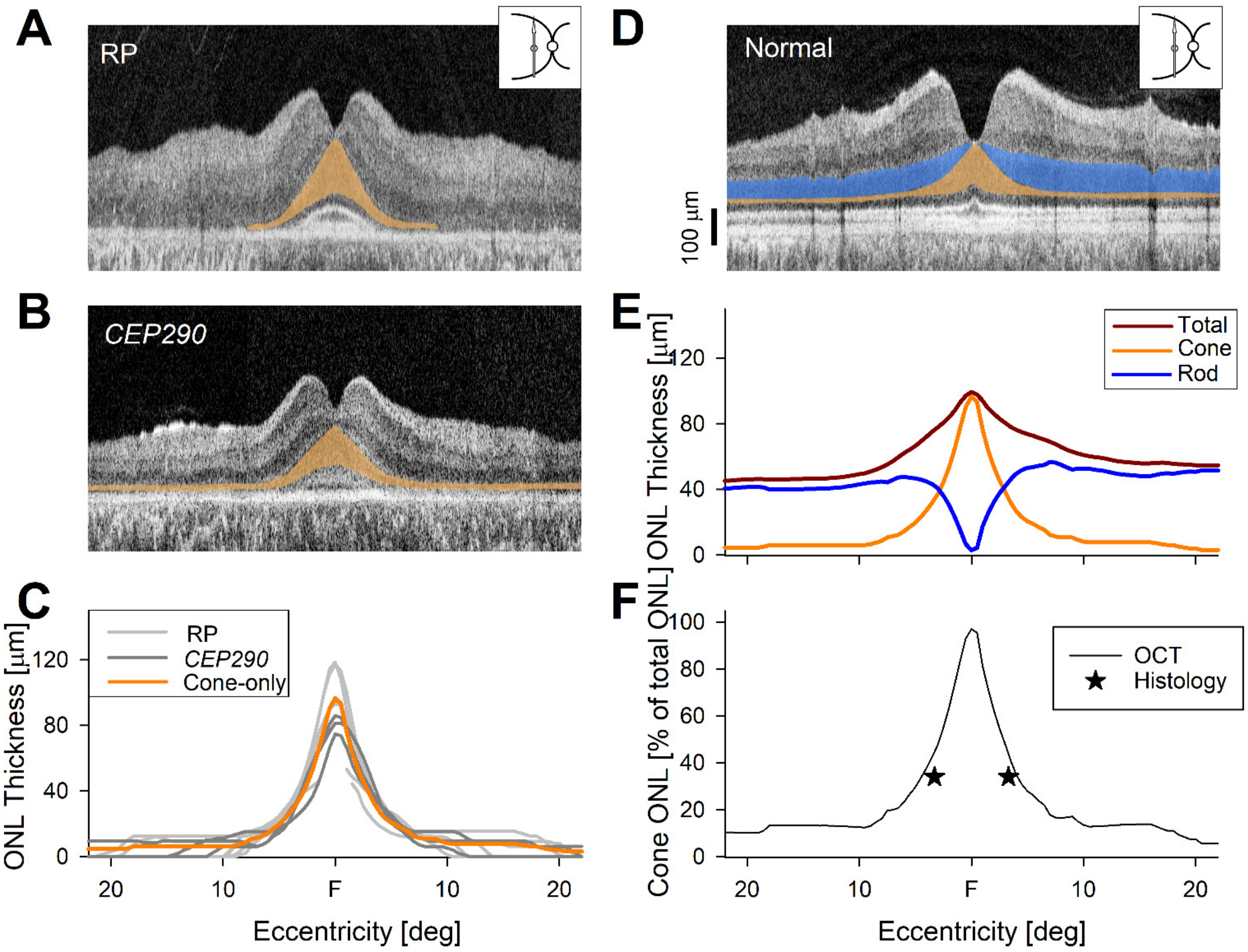


**Fig. S3.** Estimating the rod and cone components of the normal ONL. (**A**,**B**) OCT scans along the vertical meridian from representative a RP patient (A) with only cone function remaining, and a *CEP290*-LCA patient (B) with dysfunctional but retained cones. ONL layer hypothesized to contain only cone nuclei is highlighted orange. (**C**) ONL thickness along the vertical profile (gray lines) from a cohort of RP (not including *RHO*-adRP) and *CEP290*-LCA patients hypothesized to have only cone nuclei remaining. The average estimate of ONL thickness representing only cone nuclei shown (orange line). (**D**) A representative normal OCT scan along the vertical meridian. Highlighted are the estimated rod (blue) and cone (orange) components of the ONL. (**E**) Mean rod, cone and total ONL thickness estimates as a function of eccentricity along the vertical meridian. (**F**) Cone ONL thickness as a percent of total ONL thickness along the vertical meridian. Symbols are available data from the literature based on normal human retinal histology of rod and cone ONL thicknesses measured along the horizontal meridian (20).

**Table S1.** Demographics of Class B *RHO*-adRP patients with long-term followup included in Figures 1 and 2.

| | | | Long-term Function Figure 1 EF | | Long-term Function Figure 1 G | | | Long-term Structure Figure 2 | |
|---|---|---|---|---|---|---|---|---|---|
| **P#** | **Mutation** | **Eye** | **Age 1** | **Interval** | **Age 1** | | **Interval** | **Age 1** | **Interval** |
| P9 | Pro23His | RE | 18.0 | 12.3 | 18.0 | | 12.3 | 18.0 | 12.3 |
| P12 | Pro23His | RE | 15.5 | 28.3 | 15.5 | # | 28.3 | | |
| P13 | Thr58Arg | RE | 19.5 | 25.6 | 19.5 | # | 25.6 | | |
| P15 | Thr58Arg | RE | 23.9 | 25.3 | 23.9 | # | 25.3 | | |
| P16 | Glu344Ter | RE | 24.6 | 23.8 | 24.6 | # | 23.8 | | |
| P17 | Thr58Arg | LE | 26.7 | 25.8 | 26.7 | # | 25.8 | | |
| P19 | Glu344Ter | RE | 29.9 | 22.8 | 29.9 | # | 22.8 | | |
| P20 | Pro23His | RE | | - | | | | 44.5 | 9.7 |
| | | LE | 28.4 | 25.8 | 44.5 | | 9.7 | 44.5 | 9.7 |
| P21 | Thr342Met | RE | | | | | | 43.2 | 10.5 |
| | | LE | 43.2 | 10.5 | 43.2 | | 10.5 | 43.2 | 10.5 |
| P22 | Pro23His | RE | 31.8 | 25.5 | 42.2 | | 15.1 | 42.2 | 15.1 |
| | | LE | | | | | | 42.2 | 15.1 |
| P23 | Gly106Arg | RE | 38.0 | 18.7 | 38.0 | | 18.7 | 38.0 | 18.7 |
| | | LE | | | 38.0 | | 18.7 | 38.0 | 18.7 |
| P25 | Gly106Arg | RE | 43.5 | 18.6 | 43.5 | | 18.6 | 43.5 | 18.6 |
| P26 | Thr17Met | RE | 35.5 | 24.8 | 35.5 | # | 24.8 | | |
| P27 | Gly106Arg | RE | 42.3 | 18.6 | 42.3 | | 18.6 | 42.3 | 18.6 |
| P31 | Gly106Arg | RE | | | 63.8 | | 16.5 | 63.8 | 16.5 |
| | | LE | 63.8 | 16.5 | 63.8 | | 16.5 | 63.8 | 16.5 |
| P32 | Glu344Ter | RE | 28.6 | 23.6 | 28.6 | # | 23.6 | | |
| | | N= | 16 | 16 | 18 | | 18 | 13 | 13 |
| | | Avg= | 32.1 | 21.7 | 35.7 | | 19.7 | 43.7 | 14.6 |

# limited data

**Table S2.** Demographics of Class B *RHO*-adRP patients with short-term followup included in Figure 3.

| | | | Figure 3 | |
|---|---|---|---|---|
| P# | Mutation | Eye | Age 1 | Interval |
| P1 | Pro23His | BE | 8.0 | 2.0 |
| P3 | Thr58Arg | BE | 17.9 | 2.7 |
| P4 | Pro23His | BE | 21.4 | 2.0 |
| P5 | Asp190Tyr | BE | 19.8 | 2.9 |
| P6 | Thr17Met | BE | 22.0 | 2.1 |
| P8 | Pro23His | BE | 25.6 | 2.0 |
| P9 | Pro23His | BE | 28.3 | 2.0 |
| P12 | Pro23His | BE | 41.8 | 2.0 |
| P15 | Thr58Arg | BE | 47.2 | 2.0 |
| P17 | Thr58Arg | LE | 49.9 | 2.5 |
| P18 | Pro23His | BE | 50.7 | 2.0 |
| P20 | Pro23His | BE | 52.2 | 2.0 |
| P22 | Pro23His | BE | 55.4 | 2.0 |
| P24 | Pro23His | BE | 57.6 | 2.0 |
| P26 | Thr17Met | BE | 58.2 | 2.1 |
| P27 | Gly106Arg | BE | 58.8 | 2.1 |
| P30 | Pro23His | BE | 66.3 | 2.0 |
| | | N= | 17 | 17 |
| | | Avg= | 40.1 | 2.1 |

Table S3. Demographics of Class B *RHO*-adRP patients included in Figure **4**.

| | | | Figure 4D | |
|---|---|---|---|---|
| **P#** | **Mutation** | **Eye** | **Visit 1 Age** | **Visit 2 Age** |
| P1 | Pro23His | BE | 8.0 | 10.0 |
| P2 | Gly89Asp | BE | 11.0 | - |
| P3 | Thr58Arg | BE | 17.9 | 20.6 |
| P4 | Pro23His | BE | 21.4 | 23.4 |
| P5 | Asp190Tyr | BE | 19.8 | 22.7 |
| P6 | Thr17Met | BE | 22.0 | 24.1 |
| P7 | Thr17Met | BE | 24.0 | - |
| P8 | Pro23His | BE | 25.6 | 27.6 |
| P9 | Pro23His | BE | 28.3 | 30.4 |
| P12 | Pro23His | BE | 41.8 | 43.8 |
| P14 | Pro23His | BE | 43.5 | - |
| P15 | Thr58Arg | BE | 47.2 | 49.2 |
| P17 | Thr58Arg | LE | 49.9 | 52.5 |
| P18 | Pro23His | BE | 50.7 | 52.7 |
| P20 | Pro23His | BE | 52.2 | 54.2 |
| P22 | Pro23His | BE | 55.4 | 57.4 |
| P23 | Gly106Arg | BE | 54.4 | 56.8 |
| P24 | Pro23His | BE | 57.6 | 59.7 |
| P25 | Gly106Arg | BE | 60.0 | 62.1 |
| P26 | Thr17Met | BE | 58.2 | 60.3 |
| P27 | Gly106Arg | RE | 58.8 | 60.9 |
| P30 | Pro23His | RE | 66.3 | 68.3 |
| | | N= | 22 | 19 |
| | | Avg= | 39.7 | 44.0 |

## SI References


1. V. N. Highman, R. A. Weale, Rhodopsin density and visual threshold in retinitis pigmentosa. *Am. J. Ophthalmol.* **75**, 822-32 (1973).
2. H. Ripps, K. P. Brin, R. A. Weale, Rhodopsin and visual threshold in retinitis pigmentosa. *Invest. Ophthalmol. Vis. Sci.* **17**, 735-45 (1978).
3. I. Perlman, E. Auerbach, The relationship between visual sensitivity and rhodopsin density in retinitis pigmentosa. *Invest. Ophthalmol. Vis. Sci.* **20**, 758-65 (1981).
4. S. G. Jacobson, C. M. Kemp, C. H. Sung, J. Nathans, Retinal function and rhodopsin levels in autosomal dominant retinitis pigmentosa with rhodopsin mutations. *Am. J. Ophthalmol.* **112**, 256-71 (1991).
5. P. E. Kilbride, K. R. Alexander, M. Fishman, G. A. Fishman, Human macular pigment assessed by imaging fundus reflectometry. *Vision Res.* **29**, 663-74 (1989).
6. A. E. Elsner, S. A. Burns, R. H. Webb, Mapping cone photopigment optical density. *J. Opt. Soc. Am. A.* **10**, 52-8 (1993).
7. S. Marcos, R. P. Tornow, A. E. Elsner, R. Navarro, Foveal cone spacing and cone photopigment density difference: objective measurements in the same subjects. *Vision Res.* **37**, 1909-15 (1997).
8. D. Huang, *et al.*, Optical coherence tomography. Science 254, 1178-81 (1991).
9. J. Fujimoto, D. Huang, Foreword: 25 years of optical coherence tomography. *Invest. Ophthalmol. Vis. Sci.* **57**, OCTi-OCTii (2016).
10. S. G. Jacobson, *et al.*, Identifying photoreceptors in blind eyes caused by RPE65 mutations: prerequisite for human gene therapy success. *Proc. Natl. Acad. Sci. U. S. A.* **102**, 6177-82 (2005).
11. A. V. Cideciyan, *et al.*, Human gene therapy for RPE65 isomerase deficiency activates the retinoid cycle of vision but with slow rod kinetics. *Proc. Natl. Acad. Sci. U. S. A.* **105**, 15112-7 (2008).
12. C. A. Curcio, K. R. Sloan, R. E. Kalina, A. E. Hendrickson, Human photoreceptor topography. *J. Comp. Neurol.* **292**, 497-523 (1990).
13. S. G. Jacobson, *et al.*, RDH12 and RPE65, visual cycle genes causing Leber congenital amaurosis, differ in disease expression. *Invest. Ophthalmol. Vis. Sci.* **48**, 332-8 (2007).
14. S. G. Jacobson, *et al.*, Usher syndromes due to MYO7A, PCDH15, USH2A or GPR98 mutations share retinal disease mechanism. *Hum. Mol. Genet.* **17**, 2405-15 (2008).
15. S. G. Jacobson, *et al.*, TULP1 mutations causing early-onset retinal degeneration: preserved but insensitive macular cones. *Invest. Ophthalmol. Vis. Sci.* **55**, 5354-64 (2014).
16. L. M. Downs, *et al.*, Overlap of abnormal photoreceptor development and progressive degeneration in Leber congential amaurosis caused by NPHP5 mutation. *Hum. Mol. Genet.* **25**, 4211-4226 (2016).
17. S. G. Jacobson, *et al.*, Normal central retinal function and structure preserved in retinitis pigmentosa. *Invest. Ophthalmol. Vis. Sci.* **51**, 1079-85 (2010).
18. A. Sumaroka, *et al.*, Treatment potential for macular cone vision in Leber congenital amaurosis due to CEP290 or NPHP5 mutations: predictions from artificial intelligence. *Invest. Ophthalmol. Vis. Sci.* **60**, 2551-2562 (2019).

19. A. V. Cideciyan, *et al.*, Cone photoreceptors are the main targets for gene therapy of NPHP5 (IQCB1) or NPHP6 (CEP290) blindness: generation of an all-cone Nphp6 hypomorph mouse that mimics the human retinal ciliopathy. *Hum. Mol. Genet.* **20**, 1411-23 (2011).
20. C. A. Curcio, *et al.*, Human chorioretinal layer thicknesses measured in macula-wide, high-resolution histologic sections. *Invest. Ophthalmol. Vis. Sci.* **52**, 3943-54 (2011).
21. S. G. Jacobson, *et al.*, Automated light- and dark-adapted perimetry for evaluating retinitis pigmentosa. *Opthalmology* **93**, 1604-11 (1986).
22. D. B. McGuigan, *et al.*, Automated light- and dark-adapted perimetry for evaluating retinitis pigmentosa: filling a need to accommodate multicenter clinical trials. *Invest. Ophthalmol. Vis. Sci.* **57**, 3118-28 (2016).
23. A. V. Cideciyan, *et al.*, Human cone visual pigment deletions spare sufficient photoreceptors to warrant gene therapy. *Hum. Gene Ther.* **24**, 993-1006 (2013).
24. N. V. Rangaswamy, H. M. Patel, K. G. Locke, D. C. Hood, D. G. Birch. A comparison of visual field sensitivity to photoreceptor thickness in retinitis pigmentosa. *Invest. Ophthalmol. Vis. Sci.* **51**, 4213-9 (2010).
25. D. G. Birch, Y. Wen, K. Locke, D. C. Hood, Rod sensitivity, cone sensitivity, and photoreceptor layer thickness in retinal degenerative diseases. *Invest. Ophthalmol. Vis. Sci.* **52**, 7141-7 (2011).
26. S. G. Jacobson, *et al.*, Defining outcomes for clinical trials of leber congenital amaurosis caused by GUCY2D mutations. *Am. J. Ophthalmol.* **177**, 44-57 (2017).
